# Recent Advances on the Application of Layered Double Hydroxides in Concrete—A Review

**DOI:** 10.3390/ma13061426

**Published:** 2020-03-20

**Authors:** Zahid M. Mir, Alexandre Bastos, Daniel Höche, Mikhail L. Zheludkevich

**Affiliations:** 1 Institute of Materials Research, Helmholtz-Zentrum Geesthacht Centre for Materials and Coastal Research, Max-Planck Str. 1, 21502 Geesthacht, Schleswig Holstein, Germany; daniel.hoeche@hzg.de (D.H.); mikhail.zheludkevich@hzg.de (M.L.Z.); 2 DEMaC—Department of Materials and Ceramic Engineering, and CICECO—Aveiro Institute of Materials, Universidade de Aveiro, 3810-193 Aveiro, Portugal; acbastos@ua.pt

**Keywords:** layered double hydroxides, reinforcement, ion exchange, corrosion, concrete

## Abstract

The issue of chloride induced corrosion of reinforced concrete is a serious problem affecting infrastructure globally and causing huge economic losses. As such this issue has gained a considerable attention in the scientific community in the recent past. Layered Double Hydroxides (LDHs) have recently emerged as a new class of concrete-additives with a potential to increase the chloride resistance of concrete and mitigate corrosion. LDHs are clay like structures consisting of positively charged layers of cations with associated hydroxides and exchangeable anions in between the layers. Due to this charge balanced structure, LDHs possess the property of encapsulating an anion from the environment and replacing it with an exchangeable anion present in its layers. Potential applications include chloride entrapment in concrete and delivery of corrosion inhibiting anions. However, many versatile compositions of LDHs can be easily synthesized and their application as cement additives reach far beyond corrosion mitigation in concrete. This review presents a summary of recent advances on the applications of LDH in concrete. An extensive set of recently published literature has been critically reviewed and trends have been identified.

## 1. Introduction

Our planet has experienced an unprecedented population growth in the last century [1]. The rise of population has been accompanied by an increase in building infrastructure [2], mostly concrete and steel. As a consequence, the cement production industry has significantly added to global warming with a contribution of almost 5% to the global CO${}_{2}$ emissions [3]. The carbon footprint of concrete industry is getting bigger as emerging economies in Asian and South-Asian regions are focusing on a rapid expansion of infrastructure. Furthermore, obsolete or outdated production facilities/methods have also contributed to an increased environmental impact of cement production [4,5,6]. To reduce the environmental impact of cement production, it is desirable to have concrete infrastructure with longer service life. However, due to the various degradation phenomena associated with reinforced concrete structures such as steel corrosion [7,8,9,10,11,12,13,14], freeze thaw cycles [15,16], ice abrasion [17,18,19], acid attack [20,21] etc., the service life of infrastructure is considerably reduced.

Out of the above mentioned degradation mechanisms, reinforced concrete structures are particularly susceptible to chloride induced corrosion [7,22]. Chloride induced corrosion occurs in bridge decks, parking decks and pavements exposed to de-icing salts as well as in infrastructure exposed to marine and coastal environments such as offshore bridge piers etc. The rapid deterioration of such vital infrastructure can have severe economic, environmental and social implications worldwide [23]. The need of the hour is to steer concrete research towards applications of innovative low-cost materials that can increase the chloride resistance of concrete structures.

In recent times, layered double hydroxides (LDHs) have emerged as a new class of engineering materials [24,25] which can aid in the corrosion control of concrete structures and potentially prolong their service life. LDHs are clay-like powdered materials which are often referred to as nano-containers or nano-reservoirs and have the ability of entrapping ions from the environment e.g., Cl^−^ ions. Potential applications include chloride ion and carbonate ion entrapment in concrete. This work provides an overview on the state-of-the-art on the applications of LDHs in concrete technology, based on a critical review of recently published reports and articles. One of the first reviews on application of LDHs in concrete was provided by Raki et al. [26] in 2004 and Yang et al. [27,28] in 2013. Since then many research groups and companies around the globe have started to work extensively with LDHs. Therefore a lot of applications of LDH can be found across various branches of concrete technology. The authors have attempted to review cited literature up to the end of year 2019. The paper presents an understanding of corrosion processes in concrete, chloride binding aspects as well as recent advances made in the applications of LDHs in concrete. The chloride binding aspects of LDH in concrete are critically reviewed but other related effects such as influence of LDH addition on mechanical properties, dosage, effect on microstructure etc. are also discussed.

## 2. Chloride Induced Steel Corrosion in Concrete

Concrete is the most widely used engineering material [29,30] which is prepared by mixing together cement binder, fine and coarse aggregates and water. After the mixing phase the concrete is able to take any desired shape upon hardening. The resulting concrete is very good to resist compressive stresses but does not display similar capabilities under tensile loading. To improve this, steel reinforcement is embedded in the concrete which is able to take tensile stresses and also provide confinement to concrete. Concrete itself being non-metallic in nature is not susceptible to corrosion, however it is the embedded metallic steel that is susceptible to corrosion and can lead to structural failure under prolonged exposure in corrosive environments.

**Figure 1:**
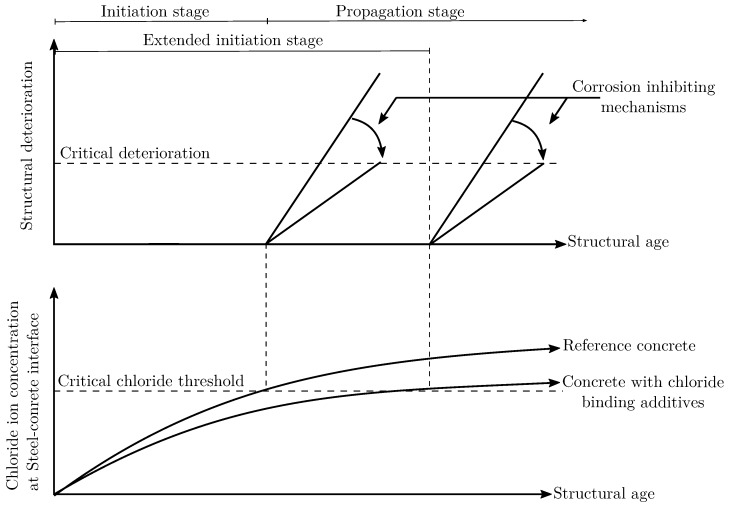
Schematic representation of service life stages of concrete structures under chloride induced corrosion and associated chloride profiles based on modified Tuutti’s diagram [7,40].

Concrete is an alkaline environment [31,32,33] and the alkalinity has a protective effect towards embedded steel bars as it helps in developing a thin protective layer on the steel surface, usually referred to as the passive layer [34,35]. This passive layer is very thin, usually a few nanometers in thickness [36] and protects the steel rebar from corrosion. At this stage, the rebar is said to be passivated and the structure is said to be in the initiation stage [7]. The porous nature of the concrete [37] allows chloride ions to pass through. In due time, chloride ions reach the steel concrete interface [38]. An accumulation of chloride ions on the rebar surface beyond a critical threshold can destroy the passive layer. At this stage the rebar is said to be depassivated [39] and the structure is said to be in its propagation stage, as shown in Figure 1. The amount of chloride ions which can cause depassivation of steel is termed critical chloride threshold [40]. A faster ingress of chloride can shorten the initiation stage of the structure and accelerate depassivation stage leading to shorter service life of structure. On the other hand, a slower or reduced ingress of chlorides can increase the service life of structure. Innovative cement additives that can slow down the chloride ingress can lead to an extension of the initiation stage. Similarly, corrosion inhibitors can reduce the severity of corrosion and mitigate structural deterioration, see Figure 1.

Once the critical threshold is reached at the steel-concrete interface, the onset of corrosion is said to take place. This leads to the formation of active anodic regions, and in the presence of moisture and oxygen lead to the formation of a corrosion cell between anodic and cathodic parts of the rebar. As corrosion progresses, rust products are generated as a result of corrosion reactions. Rust products are more expansive than parent steel [41] and their accumulation at the steel-concrete interface generates considerable internal pressure leading to micro-cracking of concrete [42,43]. As time progresses, microcracks coalesce to form visible macro-cracks which eventually can lead to the deterioration of structural elements [44].

## 3. Chloride Binding in Concrete

The transport of chloride ions in concrete matrix occurs through the pore network [45,46]. It is widely accepted that a considerable fraction of chloride ions are physically and chemically bound to the surrounding cementitious matrix [47]. These chlorides are usually named in the literature as bound chlorides. The rest of the chlorides are termed free chlorides and move freely inside the concrete pore system. It is the accumulation of free chloride on the steel concrete interface that is responsible for corrosion initiation [48]. On the other hand, the capability of concrete to bind chloride ions as well as ratio of bound chlorides to free chlorides are vital parameters regarding performance and service life estimation. Increasing the amount of bound chlorides in concrete can help in extending the time to reach the critical chloride concentration [40] on the steel concrete interface (Figure 1).

A significant contribution of bound chlorides is attributed to tricalcium aluminate (C${}_{3}$A) and tetracalcium alumino-ferrite (C${}_{4}$AF) content in binders [49,50] as they lead to the formation of aluminate monosulphate (AFm) phases upon hydration. AFm is able to chemically bind chlorides in concrete [51,52] forming Friedel’s salt which can be chemically expressed as 3CaO·Al${}_{2}$O${}_{3}$·CaCl${}_{2}$·10H${}_{2}$O or its iron analogue 3CaO·Fe${}_{2}$O${}_{3}$·CaCl${}_{2}$·10H${}_{2}$O. Depending on the concentration of chloride ion, other salts may be formed such as Kuzels salt 3CaO·Al${}_{2}$O${}_{3}$·0.5CaCl${}_{2}$·0.5CaSO${}_{4}$·11H${}_{2}$O [53]. Florea et al. [51] quantified the chloride binding of different AFm phases such as monosulphate-AFm (3CaO·Al${}_{2}$O${}_{3}$·CaSO${}_{4}$·14H${}_{2}$O) and hydroxy-AFm (3CaO·Al${}_{2}$O${}_{3}$·CaOH${}_{2}$·12H${}_{2}$O) and observed different binding capacities depending upon external Cl^−^ level.

On the other hand, the physical binding of Cl^−^ in terms of surface adsorption occurs on the calcium silicate hydrate (CSH) interlayers by replacement of loosely bound OH^−^ ion by Cl^−^ thereby maintaining electroneutrality in the system. This physical adsorption mostly proceeds on the external layers of CSH layers [54]. Tang et al. [55] reported that the amount of CSH gel is mainly responsible for the amount of chloride binding [49] whereas Florea et al. [51] considered that CSH binds fewer chlorides than AFm phases. Furthermore, it is to be noted that the chloride binding capacity varies depending on the type of the cation present in the salt [56].

Additionally, supplementary cementitious materials (SCM) are added to concrete mixes to replace cement binder, usually as a replacement. One of the motivations behind replacement of cement binder by SCM is to reduce the carbon footprint of concrete industry and also to impart additional functionality to concrete [57]. As such, fly ash (FA), ground-granulated blast furnace slag (GGBFS), silica Fume(SF), metakaolin (MTK) etc. are used as SCM in concrete. Likewise, the addition of SCM also significantly effects the chloride binding properties of concrete. It is now widely accepted that chloride binding increases with the addition of FA, GGBFS [58,59], MTK [57] whereas the addition of SF reduces the amount of bound chlorides [49]. It is believed that ion exchange mechanism is responsible for the chloride binding.

In the last two decades, Layered Double Hydroxides have emerged a new class of nanoscale engineered concrete additives which can impart additional functionality to concrete. Although the role of LDHs in concrete technology is widespread in different areas, the most outstanding application of LDH is its ability to trap anions (possibly aggressive ions such as Cl^−^) from the environment and release a tailored anion in its place (like a corrosion inhibiting anion such as NO${}_{2}$^−^) via a controlled release mechanism. This property has been termed as “*Self-Protection*” of concrete and can result in an extended corrosion initiation stage as well as a reduced corrosion degradation in the propagation stage as shown schematically in Figure 1. This review highlights the most notable applications of LDH in concrete technology with a detailed insight into the chloride binding capacity of concrete with LDH.

## 4. Layered Double Hydroxides

**Figure 2:**
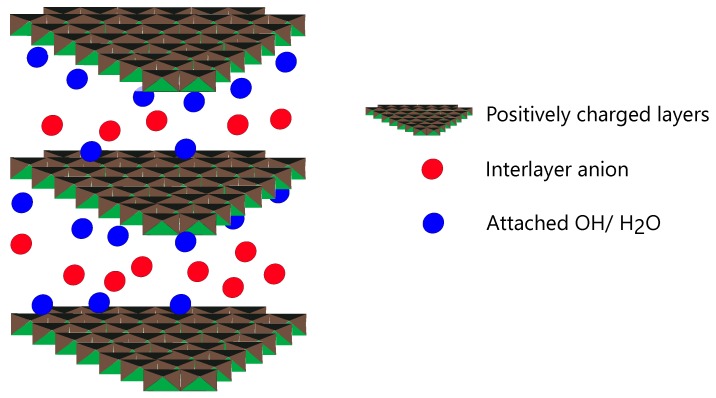
Schematic representation of Layered Double Hydroxide (LDH) structure.

Layered double hydroxides are nano-materials with a layered structure as shown in Figure 2. The layers themselves are formed of bivalent and trivalent cations coinciding with the layered structure of Brucite Mg(OH)${}_{2}$. The layers in LDHs are positively charged in nature as they are formed due to replacement of some divalent cations by trivalent cations during their formation. This leads to a charge imbalance and in order to compensate this positive charge excess, the interlayers can hold negatively charged anions, as well as some loosely bound OH${}^{-}$ / H${}_{2}$O in between the layers. LDHs can be represented by the general formula M${}_{1-\alpha }^{II}$M${}_{\alpha }^{III}$(OH)${}_{2}$(X${}^{n-}$)${}_{\alpha /n}$·mH${}_{2}$O with α ranging from 0.2–0.33, m equal to 1−3α/2, M${}^{II}$ is the divalent cation (e.g., Mg${}^{2+}$, Ni${}^{2+}$, Zn${}^{2+}$, Ca${}^{2+}$), M${}^{III}$ is the trivalent cation (e.g., Co${}^{3+}$, Al${}^{3+}$, Fe${}^{3+}$, Ga${}^{3+}$) etc. and X${}^{n-}$ is the exchangeable anion with valency *n* present in interlayer galleries and can be CO${}_{3}^{2-}$, SO${}_{4}^{2-}$, Cl^−^, OH^−^, NO${}_{3}$^−^, NO${}_{2}$^−^ etc. The molar ratio of M${}^{II}$ to M${}^{III}$ is in the range between 2 to 4 [27,60]. The interlayer anion in LDH galleries can be easily exchanged with an anion present in the external environment. Due to this ion exchange property, LDHs are also called anionic clays [61]. This unique property makes LDH a very versatile material as they can help in sequestration of anions from the environment with a possibility of releasing a tailored anion in its places. LDH can therefore be used as a potential additive for the capture of corrosion causing species and release of corrosion inhibiting anions in cementitious environments. LDH can be synthesized in powdered form and added as a percentage of the binder content or in the slurry form wherein corrections to the total w/c ratio should be made considering the amount of water originally present in the slurry.

The ion exchange property of LDH has been used for a wide range of applications across different disciplines such as drug delivery systems [62], treatment of stomach ulcers [63], genetic science [64], removal of toxic anions from water [65,66,67,68], protective coatings against corrosion on metal surfaces [69], cleansing of sea water to obtain usable cultivation water [70], removal of pollutants [71], asphalt mixes with deicing property [72] etc. are a few noteworthy applications. In concrete technology, studies on LDH as a cement additive began towards the end of 20th century, and in recent years, LDHs have gained a considerable popularity as concrete additives.

### 4.1. Preparation Methods

LDHs can be prepared by various techniques. The easiest and commonly used is the co-precipitation method. In this method aqueous solution of desired divalent and trivalent metallic salts are slowly added into a mixer containing water and allowed to simultaneously co-precipitate in an alkaline environment. The pH is held constant by adding an alkaline solution of desired pH [73]. Usually co-precipitation is done in the pH range of 8–10 [74]. Another simple method is the ion-exchange method in which a pre-synthesised LDH precursor is modified by allowing it to conduct ion exchange of its interlayer anions with desired guest anion present in the external solution. It is important to note that the ion-exchange method is strictly influenced by the affinity of LDH towards the guest anion [75,76], the type of the exchange medium [77], temperature [78], pH value of the medium [79] as well as the chemical composition of the LDH itself [80]. Both the co-precipitation method and the ion-exchange method can be used to synthesize LDH with corrosion inhibiting ions, e.g., NO${}_{2}$^−^. Using oxide of divalent cation and salt of trivalent cation, Zuo et al. [81] was able to directly synthesize LDH-NO${}_{2}$ in an alkaline environment containing NaNO${}_{2}$. Another method of obtaining the same LDH would be to first synthesize LDH-NO${}_{3}$ and then allow the LDH to conduct ion exchange with an environment containing NO${}_{2}$^−^ ions. Both the synthesis paths provide different quantities of intercalated NO${}_{2}$^−^ in LDH [81].

Another method that is commonly followed is the calcination [82,83] method where LDH is heated to high temperatures in order to remove interlayer water/OH^−^ and interlayer anions, thereby losing it layered structures and forming amorphous metallic oxides. Upon rehydration the LDH is able to reconstruct its structure using its “memory effect” property [84] upon exposure to water and also intercalate new anions which were not present in the parent LDH [80]. Apart from these preparation procedures, other methods of synthesis do exist [85,86] and the interested reader is directed to the work of He et al. [80]. LDHs also occur freely in nature. Hydrotalcite is a naturally occurring mineral and belongs to the family of LDH, being known since the 19^*th*^ century. Chemically, it can be represented as Mg${}_{6}$Al${}_{2}$(OH)${}_{16}$[CO${}_{3}$]·4H${}_{2}$O. It was first discovered in Norway and reported by German-Austrian chemist Carl C. F. Hochstetter in 1842 [87].

LDHs are not strange to concrete chemistry either. The hydration of cement binder is accompanied by the formation of a family of hydrated calcium aluminate phases (AFm) which belong to the broader class of the LDH family. According to Matschei et al. [88], in some blended cements AFm phases content can be upto 20%. AFm phases just like synthetic LDH can be represented by the formula [Ca${}_{2}$(Al/Fe)OH${}_{6}$]·*A*·xH${}_{2}$O with *A* as the exchangeable anion [89]. AFm phases resembling LDH compounds consist of brucite like sheets with some divalent cations replaced with trivalent cations such as Fe${}^{3+}$ or Al${}^{3+}$. Similar to LDH, these AFm show anion exchange capabilities with more affinity to divalent anions such as CO${}_{3}^{2-}$, SO${}_{4}^{2-}$ etc. than mono-valent anions Cl^−^, OH^−^ etc. Additions of ground granulated blast furnace slag (GGBFS) as SCM also leads to the production of hydrotalcite like phases [90,91] which has chloride binding properties [92]. Studies of Kayali et al. [93] revealed hydrotalcite formation exists in cement blends with GGBFS. Formation of hydrotalcite lead to reduced chloride ingress due to ion exchange capabilities of these LDH.

### 4.2. Ion Exchange Property Of LDH

**Figure 3:**
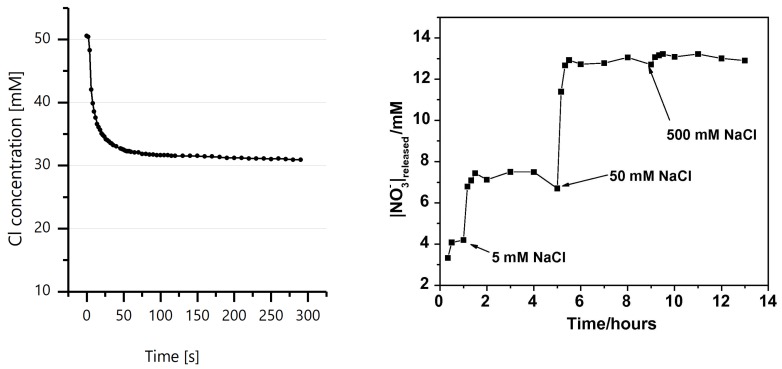
(**Left**) Decrease in chloride ion concentration due to addition of Zn-Al-NO_2_ to the solution containing 50mM of initial chloride concentration. (**Right**) Release of NO${}_{3}$^−^ ion from Zn-Al-NO${}_{3}$ after exposure to Cl^−^ ion (reprinted from [94] with permission from Elsevier).

As mentioned previously, the ion exchange property is the most vital property concerning the application of LDH in concrete. Figure 3 (left) depicts the ability of LDH to capture ions from the environment. In this test 1g of LDH was exposed to 50 mM of Cl^−^ containing solution at time (t = 0 s) under stirring conditions. Chloride ion concentration was constantly monitored for 300 s after LDH immersion. It is evident that LDH was able to reduce the Cl^−^ concentration from the surrounding solution. Figure 3 (right) depicts the ion release property of LDH. In this work from Tedim et al. [94] release of NO${}_{3}$^−^ ion from Zn-Al-NO${}_{3}$ due to step wise exposure to Cl^−^ ions is presented. This confirms the ion release property of LDH. An in-depth explanation on the ion-exchange property is presented later. Apart from the ion exchange property, LDHs posses numerous other interesting properties which are of vital importance in other branches of science and technology. The interested reader is referred to the extended literature on LDH by Evans et al. [74], Forano et al. [95] and Kuang et al. [96].

## 5. ompatibility of Ldh with Cementitious Environments

LDHs have been mostly used in concrete in order to improve its chloride resistance by harnessing the ion exchange property of LDH. However, prior to discussing the ion exchange property and chloride encapsulation of Cl^−^ by LDH in cement/concrete, it is important to understand the compatibility of LDH with cement. In this section, compatibility with cement, influence on mechanical properties and the effect of dosage on the microstructure are discussed. The discussion is based on a critical analysis of recently published literature in this field.

### 5.1. Effect on Mechanical Properties

LDH are nanoparticles which are reported to refine the microstructure of concrete without causing a substantial increase in porosity. As LDH is usually added in its powdered form to concrete mixes, these nanoparticles are uniformly distributed in the matrix and in the course of cement hydration process, LDH can provide additional nucleation sites for cement hydration. This in turn can promote growth of CSH gel into the voids, thereby refining the microstrucutre. Additionally, the particles themselves act as micro-fillers or micro-aggregates inside the cementitious matrix. Depending on the type of LDH in use, it can have a positive effect on the mechanical properties of concrete especially its compressive strength, see Table 1. LDHs have hexagonal platelet like structures and if they are able to retain their structure in mature concrete, the resulting concrete can also show enhanced flexural strength as these thin LDH platelets can act as micro-beam elements [97] between cementitious materials and therefore help in an efficient transfer of bending stresses. Table 1 presents a compilation of compressive and flexural strength enhancement of concrete together with type of LDH and dosages used by different authors.

**Figure 4:**
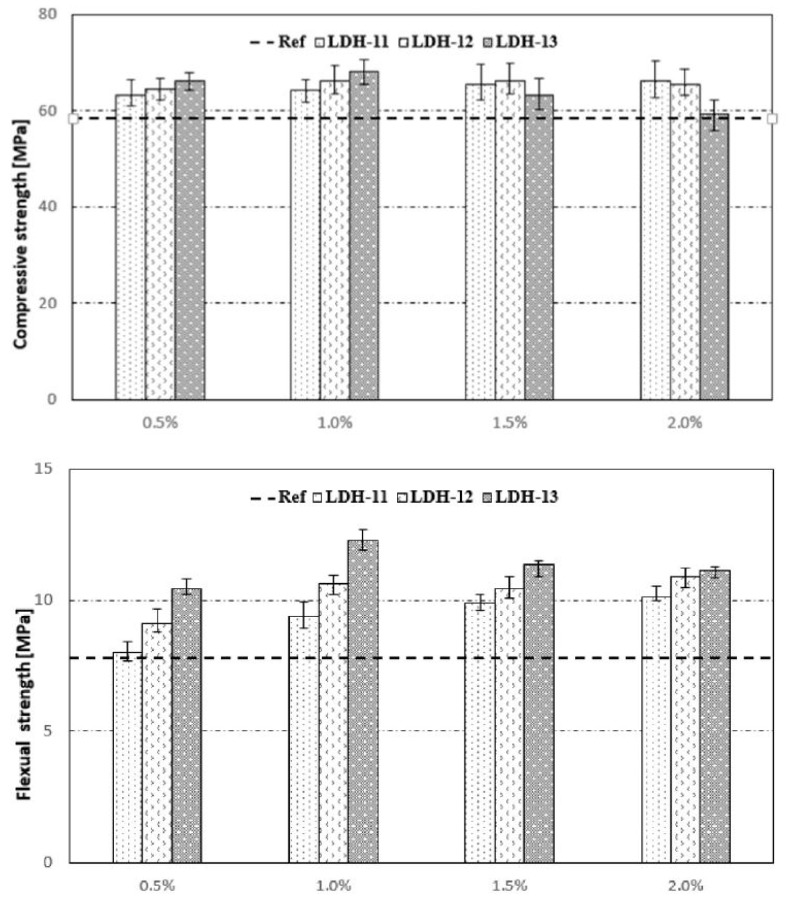
LDH dosage and effect on compressive and flexural strength. LDH-11, LDH-12, LDH-13 refer to mean particle size of 9.5 μm, 6.3 μm and 3.2 μm respectively. Reprinted from [97] with permission from Elsevier.

From Table 1, it can be observed that Ca based LDH has a profound positive effect on mechanical properties whereas multiple entries report a Mg based LDH to have a negative or inconclusive effect on mechanical properties. Qu et al. [97] used Ca based LDH and observed a strong increase in the compressive strength and flexural strength in samples with LDHs as compared with reference samples. A 17.2% increase in compressive strength was observed in samples with 1% dosage of LDH (mean particle size 3.2 μm) but only 1.7% strength increase for 2% LDH, see Figure 4. It can be inferred that a dosage of up to 2% could be safe for application in concrete and will not drastically affect the compressive strength. Higher dosages can lead to agglomeration of the particles and may not play any significant role in enhancing the strength of concrete. The particle size is also important as smaller particles can have a filler effect and refine microstrucutre leading to higher compressive strengths despite only a slight change in porosity. In the same study, the flexural strength increased with addition of LDH. The particle size was also a dominating factor as finer the particle, higher was the strength increase. However, as dosage of LDH (mean particle size 3.2 μm) increased from 1% (+55% increase) to 1.5% (+44.3% increase) and 2% (+40.5% increase), the flexural strength started decreasing although still considerable higher ∼>40% than reference sample, see Figure 4. The authors concluded that overall dosages up to 2% can have a positive influence on mechanical properties of concrete. Higher dosages than 2% can be the threshold for particle agglomeration [97].

Contrary to the studies of Qu and colleagues [97], Yang et al. [100] used Mg based LDH (see Table 1) and reported a trend of decreasing compressive strength as dosage of LDH increased, for all specimens at all ages. The strength decrease was still in the acceptable range in samples with up to 5% LDH. The negative effect on compressive strength due to addition of Mg LDH was also reported in the studies of Duan et al. [99]. However the results were found to be inconclusive as with 1% LDH, the compressive strength slightly increased but remained almost the same with 2% LDH. In another study, Xiong et al. [101] used calcined Mg-Al-LDH and reported a reduced compressive strength for mortars with LDH. A 28d strength test revealed a decrease of about 25% in the compressive strength. Studies by Yoon et al. also reported a slight decrease in compressive strength [82].

### 5.2. Effect of LDH Dosage on Microstructure

Different authors who have worked with LDH have resorted to different dosages of LDHs for applications in concrete. Usually a range of values is evaluated and higher dosages are used with the aim of improving a desired property of concrete or to check the ultimate threshold of the dosage in relation to the desired property. However, while one property might improve, addition of LDH can affect other important properties of mortars and concrete. One of the predominant effects is on porosity as found by Yang et al. [100] where porosity increased with the increasing dosage of LDH. This can have detrimental effect on other associated concrete properties such as chloride penetration. Increase of porosity can lead to faster ingress of chloride ions upon exposure and shorten the corrosion initiation stage. As the porosity increases the chloride ingress can increase despite high chloride binding. It is important to emphasize that more parameter studies must be conducted while selecting a suitable LDH dosage for applications in concrete, as dosage can have a big impact on the properties on concrete mixes.

Previous studies by Qu et al. [97] have revealed that low dosages of LDH do not have a strong influence on porosity increase. They observed that dosages up to 2 vol.%, are a good choice for incorporation of LDHs in concrete. Moreover, additions of nanoparticles like LDH up to a certain dosage can refine the microstrucutre without changing the total porosity which is advantageous towards chloride durability. This has also been reported by other authors working with nano-additives for concrete [106,107]. The use of LDH for pore refinement was carried out by Duan et al. [99]. They used Mg-Al-LDH in raw and calcined form in cement pastes and concretes (The dosage is presented in Table 1) and observed pore refinement due to addition of Mg based LDH.

### 5.3. Effect ON Hydration

LDHs have also been used to modify the hydration kinetics of concrete. It is generally believed that the addition of nano-particles can lower the energy barrier for precipitation reactions taking place in liquid state and also provide additional nucleation sites for cement hydration due to their very small size and very high surface area [98,108,109]. In a recent study, Xu et al. [98] observed by using XRD that adding nano-particles of Ca based LDH accelerated the formation of hydration products, notably CSH. This in turn resulted in a increase of early age strength development. The authors used Ca-Al-Cl LDH in their study and it is well known that both Ca${}^{2+}$ and Cl^−^ are the most accelerating cation and anion respectively [110]. One can therefore attribute the accelerating effect of LDH addition to the role of Ca${}^{2+}$ and Cl^−^ ions. The underlying mechanisms regarding effect of various ion on the hydration kinetics are not fully understood and the reader is directed to the works of Myrdal [110] for further reading.

**Figure 5:**
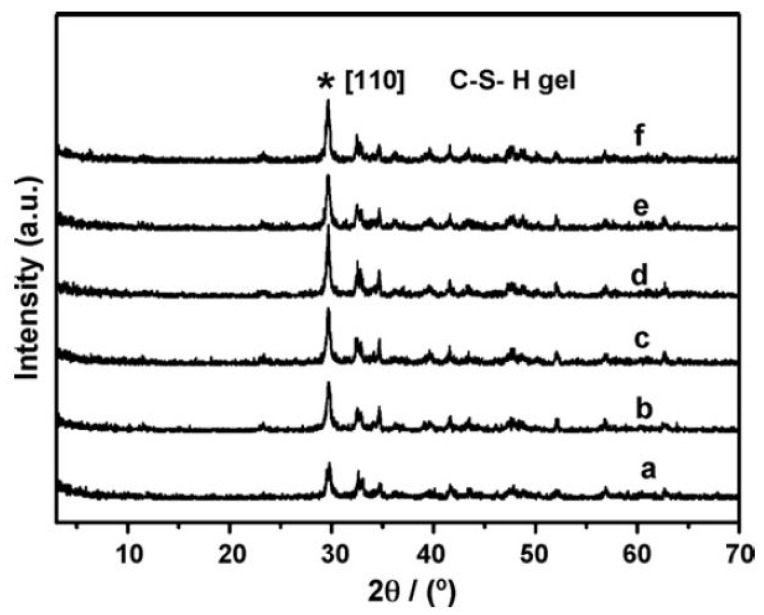
In situ XRD patterns of cement mortar (a) without and (b–f) with LDHs additives after different aging times: (b) 6 min; (c) 1 d; (d) 3 d; (e) 7 d; (f) 28 d. Symbol of * shows the characteristic [1 1 0] reflection peak of C–S–H gel. Reprinted from [98] with permission from Elsevier.

Additionally, Xu et al. conducted in situ XRD analysis of cement mortars with and without Ca${}^{2+}$ based LDH and observed that higher amounts of CSH gel are formed at early ages confirming the strength increase effect and hydration acceleration property of Ca-Al-Cl LDH. XRD analysis revealed higher intensity of CSH peaks occurring at early ages and this effect was amplified as concrete aged, see Figure 5. Li based LDH can also potentially be used to accelerate hydration [105,111] and impart early strength to concrete although the mechanism is unclear. Haiyan et al. [102] observed that setting time was reduced to almost half by using 3% addition of Li based LDH but they did not comment on the working mechanism. Moreover the authors also reported a sharp increase in the early strength development for samples with Li based LDH, see Table 1. Mg-based LDH have also been reported to have a slight accelerating effect on cement hydration. In the studies of Wu et al. [103], the authors observed that a 3% Mg LDH addition had an accelerating effect on cement hydration. They attributed this effect to the reaction of sulphate ions from gypsum with LDH that could potentially accelerate cement hydration.

On the other hand, LDH can also decelerate or retard cement hydration. Recent studies by Gomes et al. [25] reveled that addition of ZnAl LDH had a decelerating effect on hydration processes and early strength development. They used a dosage of 2–5% LDH to cement with an average particle size of 25 μm in cement pastes. The samples depicted an unusually long hardening time. The authors attributed this effect to the partial dissolution of ZnAl LDH in high alkaline pH which releases Zn${}^{2+}$ ions in to the pore solution. Zn${}^{2+}$ ions are known to have a strong retarding effect on cement hydration [112,113]. The presence of Zn${}^{2+}$ ions in the early stages of cement hydration can lead to formation of Zn-Ca complexes such as Ca(Zn(OH${}_{3}$)${}_{2}$)·2H${}_{2}$O which in turn reduce the concentration of Ca${}^{2+}$ and OH^−^ in the pore solution thereby retarding the hydration reactions [114]. Gomes et al. [25] also observed that particle size matters because bigger agglomerates ∼125 μm did not affect the hydration. This can be understood considering the reduced surface area of bigger agglomerates which do not suffer so significant dissolution as compared to finer particles.

## 6. Ion Exchange Property and Self Protection of Concrete

**Figure 6:**
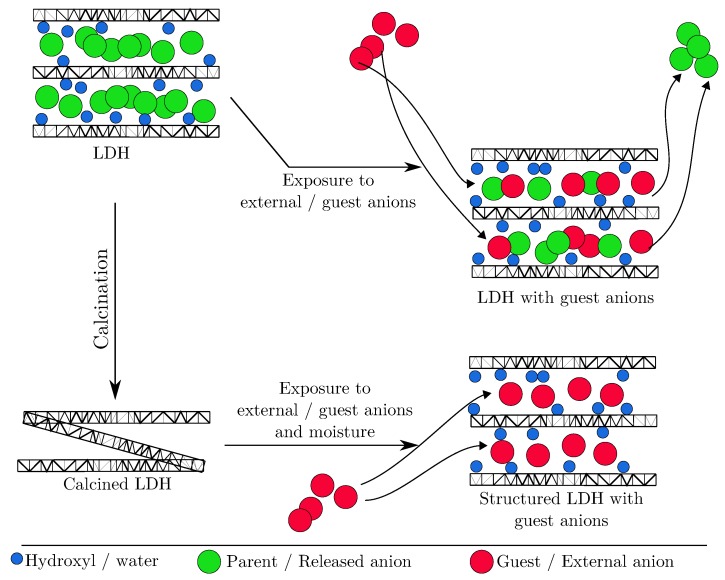
Schematic representation of the LDH structure and ion exchange/capture mechanism.

LDHs by their nature and unique chemical structure are able to exchange anions present in the external environment by an anion from their inter layers. This process is governed by the difference of chemical potential in the direction of decrease of Gibbs free energy. In order to maintain electro-neutrality, a charge balance is maintained before and after the ion exchange [26,27,81,94,115,116,117,118,119,120,121,122,123,124]. This property has been termed as the ion exchange property of LDH [122]. As such LDHs can act as anion adsorbents or anion scavengers, in different environments [125]. Furthermore, as LDHs can be synthesised with different cations and exchangeable anions, this makes them a very versatile class of materials which can be tailored to deliver a desired anion and capture selective ions from the environment. Figure 6 shows a schematic representation of the structure of LDH with interlayer anions. Upon exposure to external anions, the LDH is able to capture these anions and the process is accompanied with the release of interlayer anions. This property has been exploited in corrosion mitigation of concrete structures where functional LDH is used to conduct chloride ion capture and simultaneously deliver a corrosion inhibiting anion. Not only does this prolong/extend the corrosion inhibition stage but also protects the steel from initiating corrosion processes. This dual benefit has been termed as *self-protection* process in concrete.

The ion exchange property of LDH is also used to produce LDH with a desired anion by exposing LDH to a solution of this anion. The desired anion is captured by LDH by replacing the anion originally present in its galleries. The LDH is then usually centrifuged and separated from the filtrate, washed and dried. It must be noted that anion uptake is governed by many factors such as temperature [78], particle size [97], anion type [126,127], anion charge and size [128,129] etc.

Calcined LDH has also been used to conduct ion capture in concrete [103]. Calcination of LDH results in the collapse of the layered structure of LDH. In this process the interlayer anions and the attached OH^−^/H${}_{2}$O molecules are lost. Upon exposure to moisture and external anions, the LDH is able to regenerate its layered structure with new guest anions and water molecules as shown schematically in Figure 6. The use of calcined LDH in concrete is best demonstrated by the work of Duan et al. [130] where authors used calcined Mg LDH to increase the carbonation resistance of concrete and reported very promising results.

**Figure 7:**
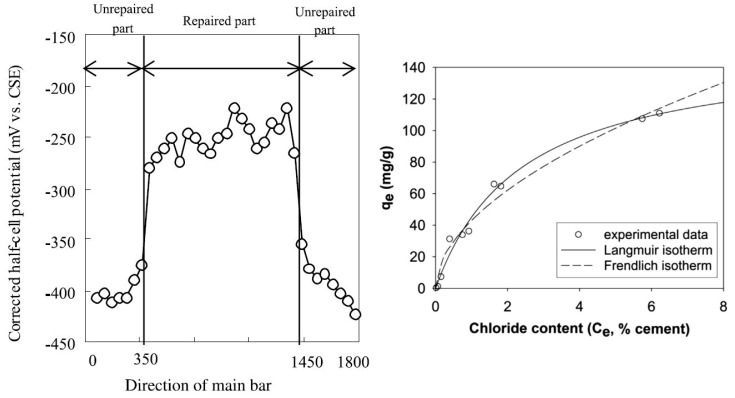
(**Left**) Figure shows steel with protective potential in the repaired part containing LDH with corrosion inhibitor (reprinted from [133] with permission from Elsevier). (**Right**) Experimental and fitted isotherms, both Langmuir and Freundlich for chloride uptake by LDH in cement paste (reprinted from [82] with permission from Elsevier).

During the ion exchange process, the ion uptake is governed by thermodynamics as it represents a heterogeneous equilibrium between chloride ions present in solid species (LDH) and the Cl^−^ ions present in the pore solution at equilibrium. Studies report that higher the external Cl^−^ ion concentration, higher is the Cl^−^ binding due to LDH. As such, the binding behavior can be represented by Freundlich or Langmiur type isotherms (Figure 7 right). In both these isotherms, uptake increases as free chloride ion concentration increases. Furthermore, for each type of LDH, its ion exchange property is governed by many factors. The most important factor ruling ion exchange is the selectivity series for that LDH. LDH is able to conduct preferential ion exchange based on the order of ions in the selectivity series. Chen et al. [131] concluded that for CaAlNO${}_{3}$ LDH in alkaline environment, the selectivity series is Cl^−^ > OH^−^ > NO${}_{3}$^−^ whereas Costa et al. [127] computationally derived the selectivity series for ZnAl LDH to be CO${}_{3}^{2-}$ > OH^−^ > F${}^{-}$ > Cl^−^ > Br${}^{-}$ > NO${}_{3}^{-}$. Although, in general the selectivity series governs the ion exchange, the series order might change if the concentration of one of the non-preferential anions is too high in the external environment as compared to the preferred anion in the series. According to the selectivity series, CO${}_{3}$^2−^ions are very stable inside LDH and difficult to replace by ion exchange. However, studies by Iyi et al. [60] concluded that LDH containing carbonate ions can even be de-carbonated by a non-preferential anion such as Cl^−^, if Cl^−^ is present in a very high concentration. In this case, the anion present in high concentration can potentially force its uptake. The size of the anion also governs ion uptake as Iyi et al. [60] was not able to replace carbonate ion using a high concentration of iodide ions. The ion exchange property of LDH prefers smaller sized anions over larger anions. However, as the ion exchange is based on charge conservation, LDHs are particularly affine to divalent anions as compared to monovalent anions with carbonate anions being the most preferred anion for most of the LDHs. As such, LDHs have been used to increase the carbonation resistance of concrete [132] as they can efficiently capture carbonate ions.

### 6.1. Chloride Entrapment and Corrosion Performance

The ion exchange property of LDH has been used extensively in the corrosion protection of concrete. Many authors have used LDH to conduct additional chloride binding in concrete as well as controlled delivery of corrosion inhibiting anions. Numerous applications also report on the use of LDH for mitigation of carbonation induced steel corrosion in concrete. Table 2 presents a list of recent works where LDH has been used an as anion exchanger focusing either on capture of chloride or carbonate ion, as well as delivery of corrosion inhibitor. Based on a detailed literature study summarized in Table 2, only a few of the reported studies are conducted in concrete and literature reports a majority of studies in salt solutions and concrete pore solutions. Table 2 also presents the preparation methods, target application exploiting ion exchange property as well as LDH dosages used. This table can aid researchers and engineers to decide on starting materials and dosages of LDH. Moreover, it is interesting to note the disparity in dosages that different authors have used in various investigations. The reported dosage varies from 0.2 wt.% [133] to 10% [100]. Attention must be paid to avoid high LDH dosage as it can strongly affect the properties of mortars and concrete, as mentioned earlier. Higher dosages can indeed increase the chloride binding capacity as more LDH will be present to capture Cl^−^ but on the other hand, higher dosages can certainly increase the porosity of concrete which facilitates Cl^−^ ingress.

LDH is able to capture Cl^−^ ions from the pore solutions lessening its concentration. Zhonghe et al. [134] studied Mg-Al-NO${}_{3}$ and Mg-Al-CO${}_{3}$ LDH and observed a higher chloride binding capacity when LDH was present. The binding capacity was lower with the carbonate based LDH because this ion is difficult to replace. Yoon et al. [82] used commercially available Mg- Al LDH and calcined it at 450 ${}^{\circ }$C. The resulting LDH was applied as a chloride entrapping additive with a 8.5 wt.% dosage in cement pastes and the authors reported enhanced chloride uptake capabilities. Based on their results, the authors confirmed the positive benefits of LDH in increasing the service life of concrete structures.

Although chloride capture due to LDHs has been mostly due to the ion exchange property, other binding mechanisms do exist. Chen et al. [131] suggested that apart from ion exchange, LDHs are able to bind Cl^−^ ion following a mechanism of dissolution and recrystallisation, similar in behavior to AFm phases in concrete. The authors concluded that the ion exchange mechanism is the major contributor but it is hard to distinguish between these mechanisms in cementitious environment. The selectivity series were demonstrated to be Cl^−^> OH^−^> NO${}_{3}$^−^. Surface adsorption of ions on LDH surface has also been reported as an uptake mechanism for LDHs. Ke et al. [83] concluded that for hydrotalcite phases in concrete, which also belong to the LDH family, surface adsorption of anions could contribute to 90% uptake capacity and the rest was attributed to ion exchange.

One of the best real life tests on the use of LDHs in concrete structural members was carried out by Tatematsu et al. [133]. In their study, a concrete slab was used as a test specimen. A central portion of the slab was retrofitted with a reinforcement cover made of mortar with Ca-Al-NO${}_{2}$ as additive. They applied LDH as a 1 mm coat on rebars and then added LDH with mortar to form a 15 mm cover on top. Other parts of the specimen did not contain additive. The specimen was left in an exposure site for seven years. After seven years, the regions with Ca-Al-NO${}_{2}$ showed no signs of corrosion and more positive potentials whereas the surrounding regions were corroding as shown in Figure 7 (left). Secondary reinforcement was also affected in the non-protected areas. The authors observed a macro-cell on the interface of protected and non-protected areas.

As mentioned previously, AFm phases in concrete also belong to LDH family and are responsible for the majority of the chloride binding [49,51,135,136]. The exchange mechanism is mostly believed to be ion exchange [135,137]. AFm phases just like synthetic LDHs lead to the formation of Friedel’s salt and Kuzel’s salt upon exposure to chloride [53,138]. The conversion of Hydroxy-AFm to Friedel’s salt upon exposure to NaCl was stated to be a dominating chloride uptake mechanism by Jones et al. [139]. The Cl^−^ uptake was observed to be followed by a delivery of OH^−^ ion from AFm phases [140]. Hirao et al. [141] experiments on chloride uptake reveled a Freundlich type isotherm for chloride uptake, signifying that uptake capacity would improve as the external concentration increases. It can therefore be concluded that AFm phases in general contribute considerably to the inherent chloride binding capacity of concrete via ion exchange mechanism [142].

The dual benefit of self-protection of reinforced concrete can be achieved by the capture of Cl^−^ ion and simultaneous release of a corrosion inhibiting ion. LDHs can be easily loaded with corrosion inhibiting ions. The use of NO${}_{2}$^−^ as a corrosion inhibitor has been well documented [143,144] and also NO${}_{2}$^−^ can be easily incorporated into LDH. However, it is important to note that the inhibitive action takes place only at the steel-concrete interface. The nitrite ions released in the bulk will have to transport themselves to the steel-concrete interface. On the other hand, LDH loaded with inhibiting ions can also be applied as a cement slurry coating on the rebar surface prior to embedment. Yang et al. [145], applied a coating of 20 wt.% LDH on the reinforcing steel and reported an extended time regarding depassivation of steel. The combined role of chloride capture from the pore solution and inhibitor delivery can be represented as

(1) $$\mathrm{LDH}\cdots {\mathrm{Inhibitor}}^{-}+\underset{(\mathrm{Pore}\;\mathrm{solution})}{{\mathrm{Cl}}^{-}}\to \mathrm{LDH}\cdots {\mathrm{Cl}}^{-}+\underset{(\mathrm{Pore}\;\mathrm{solution})}{{\mathrm{Inhibitor}}^{-}}$$

Use of NO${}_{2}$^−^ ion in corrosion inhibition was previously tested by Dry [146] who used Ca(NO${}_{2}$) in concrete and reported a delay in corrosion as compared to control samples. Gomes et at. [25] used LDH loaded with NO${}_{2}$^−^ ion with the aim of capturing Cl^−^ ion and releasing NO${}_{2}$^−^ ion and reported positive results.

**Figure 8:**
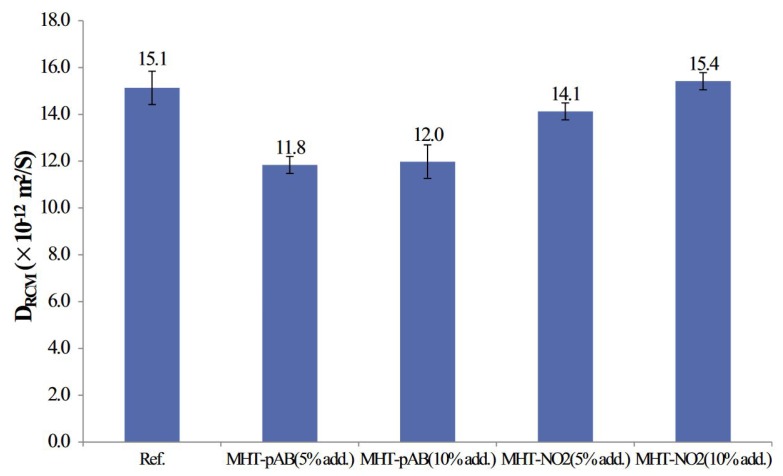
Chloride diffusion coefficients of mortar with 5% and 10% dosage of LDH. (Reprinted from [100], with permission from Elsevier).

Many authors [25,100] have conducted natural diffusion test on concrete with added LDH in order to ascertain the enhanced chloride capture in such mixes. Yang et al. [100] conducted a rapid chloride migration tests on concrete with and without LDH. Two levels of LDH were used, namely 5% and 10% for two LDH types as shown in Figure 8. Samples with 5% Mg-Al-PAB(p-aminobenzoate) demonstrated improved chloride resistance as compared to other dosages of LDH. The authors concluded that higher dosages could possible bind more chloride but at the same time also increase the porosity which has a counter effect on chloride transport in concrete.

Chloride diffusion test were also conducted by Qu et al. [97] with 0.5%, 1% and 2% by volume LDH dosage. They observed excellent chloride resistance of concrete for an optimum 1% LDH dosage. A 25% reduction of D${}_{RCM}$ (rapid chloride migration coefficient) was found for this dosage as compared to reference. The study also included natural diffusion test and demonstrated a 53% reduction in apparent diffusion coefficient. The authors explained their observations by the physical barrier properties of LDH particles acting as a filler material. This effect was complemented by chemical inhibition i.e., anion exchange property of LDH. The low dosages of up to 2% did not seem to cause any unnecessary porosity increase which is detrimental towards chloride resistance. Moreover, it was considered that smaller particles of LDH can improve the tortuosity of the cementitious matrix without actually influencing the porosity so much.

### 6.2. Role of Ldh in Carbonation Control

It is well understood that the CO${}_{3}^{2-}$ form of LDH is the most stable because LDH has a preference to multivalent ions than monovalent ions [83]. Due to this very fact, LDH has also been used to improve the carbonation resistance of concrete [147,148]. In a recent work by Ma et al. [61], cement pastes with 2% Mg based LDH showed reduced carbonation depth as compared with reference samples. They observed a 30% reduction in carbonation depth at 42 day age. Similar findings were also reported by Shui et al. [132]. This highlights the positive benefits of employing LDH in carbonation control of structures.

The high affinity of LDH towards carbonate ions can have a negative impact on chloride binding capacity of LDH. It is important to note that, if the LDH was previously exposed to Cl^−^ ions and has bound Cl^−^ ions, it might release these bound chlorides upon exposure to carbonates, since uptake of carbonate ions is preferred over chlorides [83]. Ke et al. [83] studied the chloride uptake by Mg-Al based and Strätlingite in carbonated and non-carbonated salt solutions. They reported that chloride uptake decreases as concentration of carbonate increased. Kayali et al. [58] concluded that carbonation is a risk because Cl^−^ can be replaced with CO${}_{3}^{2-}$, thereby inducing risk of accelerated steel corrosion. However under certain circumstances, LDH can be decarbonated and exchanged with other anions. Iyi et al. [60] was able to exchange Cl^−^ with CO${}_{3}^{2-}$ calling it the “decarbonation” of LDH under high concentrations of externally present Cl^−^. The study was extended to other anions as well but it was observed that only small ions such as Cl^−^ and Br${}^{-}$ are able to exchange themselves with CO${}_{3}^{2-}$. Much less or insignificant exchange was observed for larger anions such as NO${}_{3}$^−^ and ClO${}_{4}^{-}$. The anion size effect was also demonstrated by Miyata [126].

## 7. Conclusions

In this study a review on the applications of LDH in concrete is presented. The main advantage of using LDH in concrete is to improve the chloride and carbonation resistance of concrete. This can consequently extend the service life of concrete structures exposed to corrosive environments. In this aspect, this review presents many recent studies reporting on the application of LDH not only in pore solutions but also in concretes, mortars and pastes. The review also showcases that even though LDH addition is primarily aimed to improve chloride/carbonation resistance, its addition can also affect hydration, strength, microstructure and other properties of concrete. Additionally, recommendations on LDH dosage are presented. In general, this review should aid scientists and engineers to develop a basic understanding on the use of LDH as an engineering material and help in the design of experiments and be able to foresee main results. Depending of the type of LDH as starting material, quantitative and qualitative effects on desired property as well as associated properties of concrete are presented, trends are identified and selected results are discussed. A compilation of focused results are presented in text as well as in tables, highlighting the benefits of LDH as well as details of the experiments and main results.

The ion exchange property of LDH has been widely exploited in concrete technology with a majority of applications aimed towards increasing the chloride and carbonation resistance of concrete. Although the underlying mechanics for these applications are well researched and thoroughly discussed in this text, many other mechanisms are still not fully clear and vary with the type of LDH, such as, the effect of increase of compressive strength and flexural strength upon addition of LDH, as well as acceleration of hydration kinetics. Moreover, there is no general consensus on the optimum dosage of LDH in concrete, although a 2% dosage has been identified as the threshold of LDH addition from the presented literature. A higher dosage can cause agglomeration of particles and have a profound effect on key properties such as chloride durability and compressive strength.

Despite the recent advances in the application of LDH in concrete technology, many issues stay unresolved and need further understanding. Firstly, the stability of LDH particles inside concrete is not well understood and needs more research in the future. Higher pH of fresh concrete can potentially cause partial dissolution of LDH particles which can in turn reduce their functionality. Apart from that, particle size is a very important parameter as finer particles exhibit more surface area than coarse particles. It has been reported that particle size effects LDH functionality as well as some vital concrete properties. Very few studies have reported on these issues and more research is required in this area. Additionally, one important factor is the economic aspect of using LDH as a concrete additive and not much information is available on the cost of LDH and its carbon footprint. These two factors are very important for acceptance of LDH in the concrete infrastructure market. Dismantling buildings made with nano-particles can be dangerous as there is the chance that these nano particles might be released in the environment. Therefore, proper guidelines should be made available to building engineers and these buildings should be classified for easy recognition in the distant future. More research must be conducted on the interaction of humans against long term exposure to LDH. All these points need more efforts and extensive research.

However, the authors strongly believe that LDH due to its unique chemistry, versatile combinations, facile preparation and ease of incorporation into concrete, will be used more in the future to produce smart concrete structures. To make LDH a promising concrete additive, more research should be focused towards their application in concrete. One of the biggest foreseeable benefit of using LDH in concrete is the enhanced chloride/carbonate binding effect, although other notable benefits have been presented. This class of additive can result in smart structures which can potentially show extended service life and can directly result in a substantial economic benefit in the global infrastructure market.

## Figures and Tables

**Table 1:** Effect of addition of LDH on the mechanical properties of concrete. CS: Compressive strenght; FS: Flexural strength.

LDH	Cement	Dosage *	Concrete Properties		Age	Year	Ref.
Type	Type		CS	FS			
CaAl LDH	CEM 42.5	1–5%	+6% (2%LDH)	-	28d	2009	Xu et al. [98]
MgAl CO${}_{3}$	[99]	1–2%	inconclusive	-	28, 48d	2013	Duan et al. [99]
MgAl LDH	[82]	8.5%	∼−2%	-	28d	2014	Yoon et al. [82]
MgAl pAB	CEM I 42.5N	5–10%	−17.2% (10%LDH)	−21.38% (10%LDH)	28d	2015	Yang et al. [100]
MgAl NO${}_{2}$	CEM I 42.5N	5–10%	−14.2% (10%LDH)	−19.1% (10%LDH)	28d	2015	Yang et al. [100]
MgAl LDH	[101]	1%	−25% (28d)	-	28–178d	2015	Xiong et al. [101]
LiAl LDH	[102]	1–3%	+25%	-	7d	2017	Haiyan et al. [102]
CaAl NO${}_{3}$	CEM I 52.5R	0.5–2 Vol.	+17% (1% LDH)	+55% (1%LDH)	28d	2018	Qu et al. [97]
MgAl CO${}_{3}$	CEM I 52.5	1–3%	∼+3.5% (3% LDH)	-	28d	2018	Wu et al. [103]
MgAl LDH	[104]	1–2%	+8.2% (1% LDH)	-	56d	2018	Chen et al. [104]
LiAl LDH	[105]	0.5–1.5%	+46.2% (1%LDH)	-	28d	2019	Zou et al. [105]

* In mentioning the dosage, no distinction has been made for LDH added as % of binder content or % of binder replacement or % of total cementitious materials.

**Table 2:** Reported LDH types and their application in the literature together with preparation and experimental details. Abbreviations are mentioned in the end of the text. SS: Salt solution; PS: Pore solution; CP: Cement Paste; M: Mortar; C: Concrete

LDH	Experiments Conducted in					Preparation Method	Dosage ${}^{+}$		Application	Year / Ref.
	SS	PS	CP	M	C		SS / PS	CP / M / C		
CaAl NO${}_{2}$	-	-	✓	✓	-	-	-	0.2 wt.%	Cl^−^ uptake & Corr. inhibition	2003/ [133]
MgAl NO${}_{3}$	✓	-	✓	-	-	-	1 g:10 mL	1 wt.%	Cl^−^ uptake	2012 / [134]
MgAl CO${}_{3}$	✓	-	✓	-	-	-	1 g:10 mL	1 wt.%	Cl^−^ uptake	2012 / [134]
hydrotalcite	✓	-	-	-	-	calcination	8–16 g/80 mL	-	Cl^−^ uptake	2012 / [93]
MgAl NO${}_{3}$	-	-	-	-	✓	-	-	0.5–3 wt.%	Cl^−^ uptake	2012 / [149]
MgAl LDH	✓	-	✓	-	-	calcination	-	8.5 wt.%	Cl^−^ uptake	2014/ [82]
MgAl CO${}_{3}$	-	-	✓	-	✓	-	-	1–2 wt.%	Cl^−^/ CO${}_{3}^{2-}$ uptake & Pore ref.	2013 /[99]
CaAl-pAB	-	✓	-	-	-	Ion-exchange	0.5 g/10 mL	-	Cl^−^ uptake	2014 / [150]
CaAl NO${}_{3}$	-	✓	-	-	-	co-precipitation	0.5 g/10 mL	-	Cl^−^ uptake	2014 / [150]
CaAl NO${}_{3}$	✓	✓	✓	-	-	co-precipitation	1 g/100 mL	8.5 wt.%	Cl^−^ uptake	2015 / [131]
MgAl-pAB	-	-	-	✓	-	calcination-rehydration [151]	-	5–10 wt.%	Cl^−^uptake	2015 / [100]
MgAl-NO${}_{2}$	-	-	-	✓	-	calcination-rehydration [151]	-	5–10 wt.%	Cl^−^uptake	2015 / [100]
MgAl LDH	-	-	-	✓	-	calcination	-	1 wt.%	Cl^−^ uptake & bond stress	2015 / [101]
Strätlingite	-	✓	-	-	-	[152]	0.4 g/40 g	-	Cl^−^/ CO${}_{3}^{2-}$ uptake	2017 / [83]
MgAl LDH	-	✓	-	-	-	calcination	0.4 g/40 g	-	Cl^−^/ CO${}_{3}^{2-}$ uptake	2017 / [83]
MgAl NO${}_{3}$	-	✓	✓	✓	-	calcination-rehydration	0.5 g/20 mL	0–10%(M), 20 wt.%(CP)	Cl^−^-uptake & Corr. inhibition	2017/ [145]
MgAl-pAB	-	✓	✓	✓	-	calcination-rehydration	0.5 g/20 mL	0–10%(M), 20 wt.%(CP)	Cl^−^-uptake & Corr. inhibition	2017/ [145]
CaAl NO${}_{3}$	-	-	-	✓	-	co-precipitation	-	0.5–2 vol.%	Cl^−^ uptake	2018 / [97]
MgAl CO${}_{3}$	-	-	-	-	✓	calcination, other [130]	-	2 wt.%	CO${}_{3}^{2-}$ uptake	2018 / [130]
MgAl LDH	-	-	-	-	✓	calcination, other [132]	-	2–4 wt.%	CO${}_{3}^{2-}$ uptake	2018/ [132]
MgAl NO${}_{2}$	-	✓	-	-	-	Ion-exchange	1 g/10 mL, 1–2 wt.%	-	Cl-uptake & Corr. inhibition	2018 / [153]
MgAl NO${}_{3}$	-	✓	-	-	-	co-precipitation	1 g/10 mL, 1–2 wt.%	-	Cl^−^ uptake	2018 / [153]
MgAl LDH	-	-	✓	-	-	calcination	-	1–2 wt.%	Cl^−^ uptake	2018/ [104]
CaAl LDH	✓	-	-	-	✓	co-precipitation	50 g/L	10 wt.%	Cl^−^ uptake	2019/ [154]
ZnAl NO${}_{2}$/NO${}_{3}$	✓	✓	✓	-	-	co-precipitation	1 g/50 mL	2–5 wt.%	Cl^−^ uptake & Corr. inhibition	2019 / [24,25]
MgAl CO${}_{3}$	-	-	-	-	✓	calcination	-	2 wt.%	CO${}_{3}^{2-}$ uptake	2019 / [61]

${}^{+}$ In mentioning the dosage in cement paste, mortars and concrete, no distinction has been made for LDH added as % of binder content or % of binder replacement or % of total cementitious materials.

## Abbreviations

The following abbreviations are used in this manuscript:

LDH	Layered Double Hydroxide
CSE	Copper sulphate electrode
pAB	P-aminobenzoate
C${}_{3}$A	tricalcium aluminate
C${}_{4}$AF	tetracalcium alumino-ferrite
AFm	aluminate monosulphate
CSH	calcium silicate hydrate
